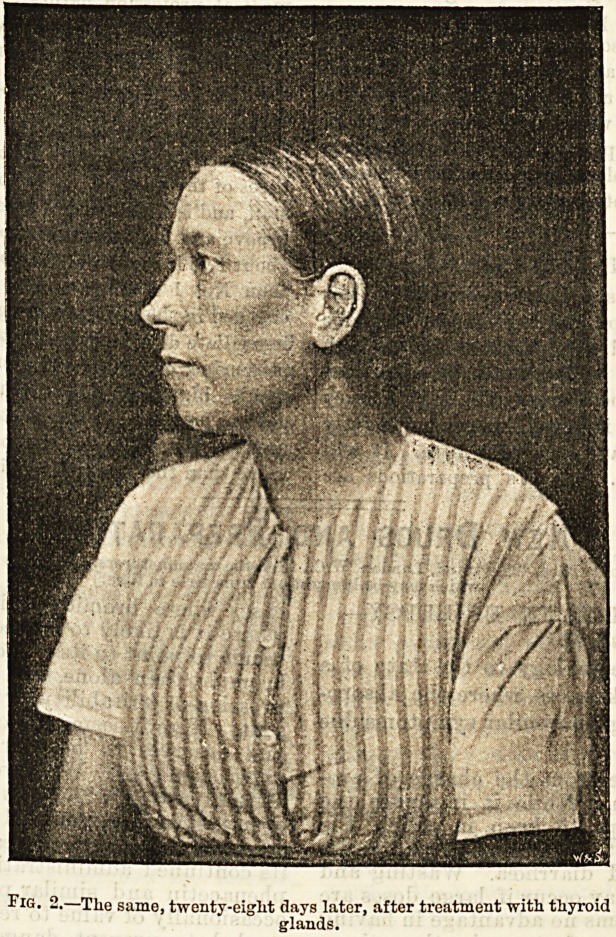# The Treatment of Myxœdema

**Published:** 1893-11-11

**Authors:** 


### THE TREATMENT OF MYXŒDEMA.

We have recently detailed the various stages through which the investigations on the relationship between the thyroid gland and myxœdema have led us, till from the comparatively prosaic fact that the disease myxœdema might be reproduced, and was frequently produced, by the extirpation of the thyroid gland in monkeys, or by the total removal of the gland from disease in human beings, we were enabled to achieve one of the most brilliant successes in medicine—the cure of myxœdema and of cretinism by administering the gland in the form of medicine.

The success in treating those affections is so marked, the disease so disastrous, not to life, but to all that makes life worth living, and the remedy so simple, that it will be difficult for future generations to realise what a splendid example of inductive reasoning as a means of devising a cure from facts gathered from the physiological laboratory and the post-mortem room, this new treatment affords. We may say truly that the method demonstrates and justifies, perhaps more completely than any other of the great achievements of modern therapeutics, the fact that we have exchanged the old "empiricism" for the new "rationalism" in the treatment of disease. We now have the means of combating the disease, resulting from a loss of functional activity of the thyroid gland. We hope to learn why the gland atrophies and loses its functional activity, and how this may be prevented.

We have likewise acquired a knowledge of the all-important fact that a condition indistinguishable from true wasting diabetes always results in animals from the complete or almost complete removal of the pancreas, and though the importance of this great fact has been discounted because we cannot just at present see how this bears on the treatment of the disease diabetes, we must remember that the treatment of myxœdema was not established till years of patient investigation had been devoted to the elucidation of a disease of the nature of which we knew much less to start with than we believe we are justified in saying we know of diabetes.

But we are reminded of the rapidity with which such an advance in therapy has been utilised by the medical profession by the very valuable contribution of Mr. Cecil F. Beadles to the "Journal of Mental Science" on the "Treatment of Myxœdema and Cretinism," with an analysis of a hundred published cases.

In only two of these hundred cases was there no improvement, and in two others only was there merely slight improvement. In many the cases were described as cured, and with the above four exceptions, the improvement, if not amounting to a cure, was very marked indeed.

One of the earlier cases treated, of which we give illustrations, was admitted to the Bristol Royal Infirmary under Dr. Watson Williams, June 13th, 1892. Thirteen months before admission S.B., female, age 48, had noticed that her sight was getting dim, and that her eyes and face were swollen, as well as her hands and neck. Her friends thought she had dropsy. After a short period her speech became muffled and thick, and her memory was very defective. She became very weak, and quite unable to work or to walk any distance. She had suffered from epileptic fits from the age of 19 up to within two years of her admission to the Royal Infirmary, they having ceased at the climacteric. It was thought that the mental torpor was partly to be attributed to the frequent recurrence of epileptic fits during the greater portion of her life, and that consequently the treatment of the undoubted myxœdematous condition would not result in such complete restoration as might otherwise have been looked for. In addition to the characteristic slow speech with low pitched voice, the solid œdema of the face, lips, hands, and the swelling in the supra-clavicular regions, the general aspect of the patient, and the defective memory, she had the usual waxy complexion, with patches of purplish injection on the cheeks, and the purple blush at the tip of the nose; and she had been losing her hair rapidly. Her pulse was slow and the temperature subnormal.

Fig. 1.—Case of Myxœdema under Dr. Watson Williams at the Bristol Royal Infirmary.

She was first fed with the raw minced thyroid glands of a sheep, taking two daily in a sandwich. After taking four the temperature ran up to 101, and the pulse rate was increased to 96 from 68 per minute. In ten days she had nineteen lobes either by the mouth or hypodermically. At the end of the tenth day the thyroid gland sandwiches gave rise to vomiting, and they were given less frequently, thus she only had nine lobes in the next nine days. The thyroid was then discontinued.

Again, nine days later, the second photograph was taken, and the improvement at the end of twenty-eight days was very striking. Not only was there a complete absence of œdema, of thickening of the lips and hands, but her general mental improvement was most pleasing to herself and her friends. She was subsequently treated with a mixture containing the extract from one lobe twice a week, and in six weeks' time was able to walk in from the country—a distance of five miles—without discomfort. At the end of a year she was as well as ever she had been for many years, although, of course, the administration of occasional doses had been continued. Such cases are only typical of the majority of the hundred that Mr. Cecil Beadles has tabulated.

Conclusions based on such a thorough analysis of the whole of the published cases are too valuable to pass unnoticed, and we therefore quote Mr. Beadles' remarks on the comparisons of various methods *in extenso.* The various methods he summarises as follows:—

1. Thyroid grafting.
2. Subcutaneous injection of an extract of the thyroid gland.
3. Injection of an extract (aqueous or glycerine) of the thyroid gland.
4. Ingestion of the thyroid gland, raw or slightly cooked.
5. Ingestion of a dry extract obtained from the thyroid gland in the form of a powder, tabloid or capsule, or pill.
6. Ingestion of thyroidin.

Of thyroid grafting he believes he had said sufficient in the body of his paper to show that so far the results obtained have, in a manner, been disappointing, and scarcely what at one time was hoped of it. They have certainly not been followed by the same striking results as those ensuing from the more recent methods adopted. At the same time, considering the fact that the treatment by these latter is not a permanent cure, and that the drug has to be taken at certain intervals in order to maintain the improved condition brought about (a fact which is easy to understand), it would seem that our only hope of a permanent cure for myxœdema lies in some method by which transplantation can be brought to greater perfection, and the graft made capable of living in its new position. Professor Horsley, at Newcastle, has lately called attention to this, when he said that "it would appear more reasonable to perform transplantation after a prefatory treatment, by feeding or injection, so as to provide that the grafted gland should be embedded in normal connective tissue, and not in diseased tissue."

Fig. 2.—The same, twenty-eight days later, after treatment with thyroid glands.

By the injection of a fluid extract subcutaneously the treatment became at once more simple and free from the many risks of a large operation. It was a small operation that no physician would mind undertaking, and its immediate effects were much more striking. Moreover, it has been shown that the cure could be maintained by the occasional use of a smaller amount than that first employed, and the ill-effects that followed its use in many of the earlier cases have been shown to be much lessened or avoided by the more careful use of the fluid, and by paying greater heed to the regulation of the dose, and the subject on whom it was being used.

A watery or glycerine extract appears to be equally efficacious, and either can be made without a great amount of trouble, although it is, perhaps, better to obtain it at regular intervals from some druggist of repute. With regard to the best dose to employ, Mr. Beadles continues, this would depend on circumstances, such as the age of the patient, duration of the disease, and various other such points that can only be decided in individual instances. Dr. Murray at the Clinical Society said he now injected ♏xv. at a time, very slowly, which caused less irritation than a larger dose, and in order to maintain the patient in health he recommended the use of a much smaller dose. In this latter, which he called the second stage of the treatment, he has also given the extract by the mouth—daily doses of ♏x. given in water—and on another occasion "he urged a small dose daily rather than a large dose at longer intervals."

Although the ingestion of thyroid glands, whether raw or slightly cooked, appears, perhaps, the simplest method possible, it is certainly not without its drawbacks. The principal of these is the difficulty of giving a fixed dose. In some cases in which this method has been employed bad symptoms have followed. When the raw glands are given they should not be more than one lobe of the thyroid two or three times a week, as recommended by Drs. Pasteur and Calvert. Dr. Hector Mackenzie does not now allow the raw gland to be eaten, as it has given rise to gastro-intestinal symptoms, but gives his patient a freshly made extract.

The use of the extract in the form of a powder is a distinct advantage in several respects. It is a grey tasteless powder, which will keep good for a sufficient period.

These are the conclusions of Dr. Beadles. Dr. Watson Williams has entirely given up the use of the extract hypodermically or by the mouth, and the crude method of administering the glands either raw or slightly cooked, using by preference the dried gland powdered and made up into pills. Each pill corresponds to one-eighth of a lobe of a young sheep's thyroid. One of these every other day is sufficient to keep the patient in good health, while in the earlier stages of treatment, when the myxœdematous condition is present, one should be given daily. No gastric disturbance has followed their administration, and the form of medication has the advantage of being that to which the public have become accustomed—further, it is convenient, simple, and inexpensive.

## Figures and Tables

**Fig. 1. f1:**
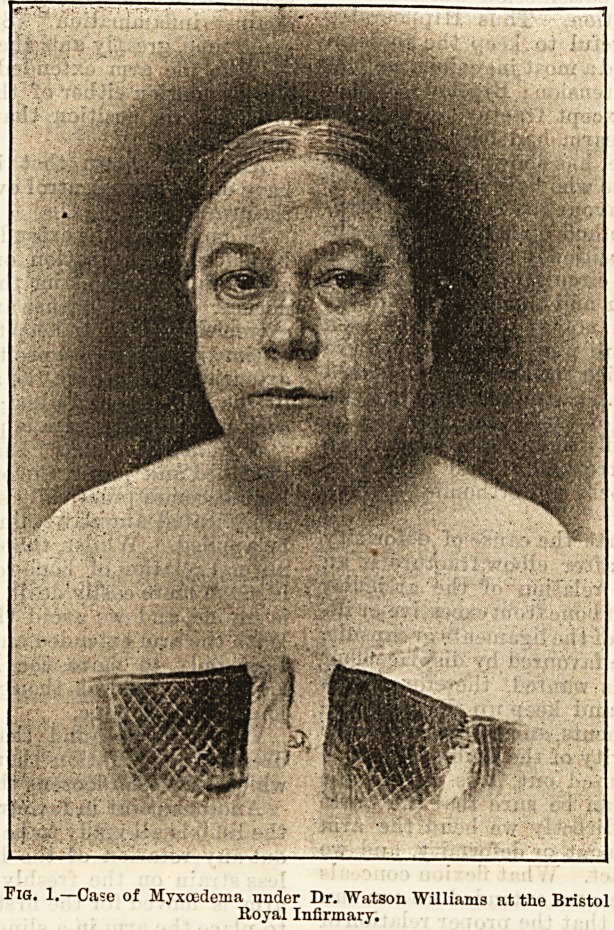


**Fig. 2. f2:**